# The New Year

**Published:** 1891-01

**Authors:** 


### HALL'S
### JOURNAL OF HEALTH

TRUTH DEMANDS NO SACRIFICE; ERROR CAN MAKE NONE.

Vol. 38. JANUARY, 1891. No. 1.

### THE NEW YEAR.

Once more the wheels of time, rolling steadily on, have completed their round of ever-recurring seasons, and brought us face to face with the hoary mile-stone that marks the passage of years; and the Journal is glad to avail itself of a time honored custom upon the threshold of the NEW YEAR, to offer its kindliest greetings and congratulations to the many friends and patrons through whose appreciative aid it has been able to sustain itself in years that are numbered with the past.

It is to be expected that of the long roll of those who have heretofore welcomed it as a monthly visitor, some will miss its presence hereafter by the family hearth-stone, or perhaps find another and possibly a more acceptable guest in its accustomed place, whilst many, very many, will still cling to the Journal as to an old and tried friend who brings to the afflicted, words of profit and good cheer.

So may it always be with THE JOURNAL OF HEALTH.

